# Progress in the application of conductive hydrogels in wound healing: a review

**DOI:** 10.1039/d5na00650c

**Published:** 2026-01-03

**Authors:** Yun Lv, Yuting Li, Yueshuai Pan, Qianqian Li, Changfang Shi, Ruting Gu, Lili Wei

**Affiliations:** a School of Nursing, Qingdao University Qingdao 266000 Shangdong China; b Department of Nursing, The Affiliated Hospital of Qingdao University Qingdao 266000 Shangdong China; c Ophthalmology Department, The Affiliated Hospital of Qingdao University Qingdao 266000 China; d Department of Spinal Surgery, The Affiliated Hospital of Qingdao University Qingdao 266000 China; e Department of Thoracic Surgery, The Affiliated Hospital of Qingdao University Qingdao 266000 China 13805424386@163.com; f Office of the Dean, The Affiliated Hospital of Qingdao University Qingdao China weilili@qduhospital.cn

## Abstract

Wound healing is a complex process in which an endogenous electrical field directs cellular migration and tissue restoration. Conventional dressings provide physical protection but cannot modulate endogenous bioelectrical signals. Conductive hydrogels address this limitation by combining the intrinsic properties of hydrogels with electrical conductivity. They not only transmit endogenous bioelectrical signals but also deliver external electrical stimulation to regulate key cellular processes such as migration, proliferation, and differentiation. The tunable properties of such materials and their adaptability to different wound environments significantly enhance their therapeutic potential. However, existing reviews focus on either specific wound types or broader biomedical applications and often lack a systematic connection between conductivity-related mechanisms and distinct wound contexts. Additionally, critical barriers to clinical translation remain understudied. This study focused on polymers suitable for conductive hydrogels, their functional mechanisms, and research advances in treating different types of wounds. Finally, it examined the key barriers to practical translation of conductive hydrogels and proposed future directions for their development as innovative wound dressings.

## Abstract 2

Wound healing is a complex process in which an endogenous electrical field directs cellular migration and tissue restoration.

## Graphical abstract

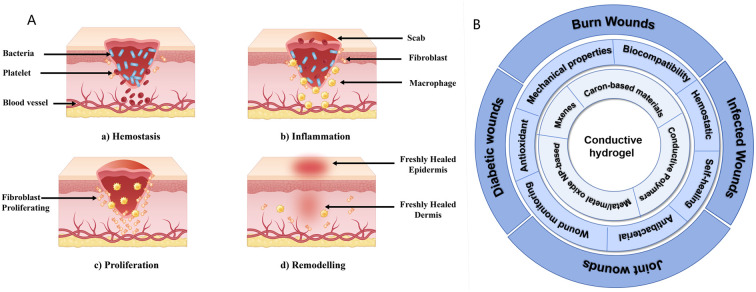

## Introduction

**Fig. 1:**
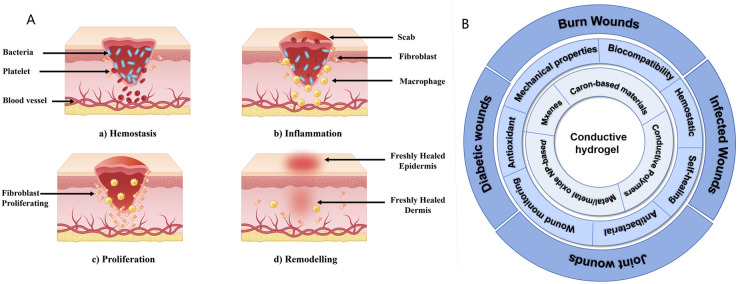
(A) Wound healing is classically divided into four stages: (a) haemostasis, (b) inflammation, (c) proliferation, and (d) remodelling. (B) Properties and applications of conductive hydrogels. This schematic illustrates the core components (innermost circle), key functional properties (middle circle), and applications of conductive hydrogels for specific wound types (outermost circle).

The skin is the largest organ in the human body and serves a vital protective function.^1,2^ It not only protects interior tissues from mechanical injury and microbial invasion but also performs physiological tasks, including sensory perception, metabolism, and electrical conduction.^3–5^ The integrity of the skin is critical to human health and safety. Once sustained damage occurs, it can result in pain, infection, and potentially life-threatening complications.^6,7^ To restore skin function and prevent these adverse outcomes, the body initiates a tightly regulated wound-healing process involving four overlapping phases: haemostasis, inflammation, proliferation, and tissue remodelling (Fig. 1A).^8^ However, the innate capacity for self-repair is often undermined by factors such as bacterial infection, excessive exudate, and chronic inflammation, emphasising the necessity for advanced wound management strategies.^9^ Effective wound treatment requires maintaining a moist environment, preventing infection, alleviating pain, and actively promoting tissue regeneration.^10–12^

Traditional wound dressings (*e.g.*, gauze and bandages) only offer passive protection and do not address the evolving needs of wound microenvironments, such as maintaining optimal moisture and supporting bioactive repair processes.^13,14^ With advancements in wound biology, there is an increasing need for novel dressings with adjustable structures and multifunctional properties.^15^ Hydrogel dressings have garnered interest as emerging options owing to their superior moisture retention, biocompatibility, customizable mechanical properties, and three-dimensional (3D) porous networks that mimic the extracellular matrix (ECM), a dynamic network of proteins and polysaccharides that provides structural and biochemical support to surrounding cells.^16–18^

Wound healing involves more than physical repair, which relies on the coordinated regulation of bioelectric and biochemical signals. Following skin injury, endogenous electric fields (40–200 mV mm^−1^) at the wound site direct keratinocyte migration, fibroblast proliferation, and angiogenesis, processes essential for effective healing.^19–21^ Disruption of these electric fields in chronic wounds (*e.g.*, diabetic ulcers) leads to delayed epithelialization and impaired tissue regeneration.^22,23^ Traditional hydrogels provide moisture retention but lack conductivity, which constrains their ability to mediate bioelectric signal transduction and amplify endogenous electrical gradients, ultimately diminishing their therapeutic potential.^24^

This identified gap has driven the development of conductive hydrogels, which integrate the intrinsic advantages of hydrogels with unique electrical properties. These materials can recapitulate physiological bioelectric microenvironments, enhancing endogenous current conduction to promote directional cell migration and vascular growth.^25–28^ Their tunable mechanical properties enable conformal contact with dynamic wound surfaces, reducing secondary trauma during dressing changes.^29^ Furthermore, their porous architecture allows the incorporation of antimicrobial agents or growth factors, enabling electrically triggered drug release to alleviate infection and inflammation.^24,30^

Previous reviews on conductive hydrogels for wound healing have frequently focused on a single wound type (*e.g.*, diabetic ulcers or burn wounds)^31,32^ or examined their biomedical applications more broadly, failing to address the distinct requirements of diverse wound pathologies.^26,33^ In contrast, this review adopts a comprehensive perspective by summarising the application of conductive hydrogel dressings across multiple common wound types and explicitly linking their material properties to the physiological needs of different wound contexts. This paper offers a comprehensive review of recent progress in conductive hydrogel dressings by examining the polymers used in their production, clarifying their key functional mechanisms, and assessing their applications across various wound types (Fig. 1B). Finally, it identifies the primary current challenges in this field and highlights critical future directions for the development of conductive hydrogel wound dressings in clinical wound care.

## Operational mechanism of conductive hydrogel

Conductive hydrogels are multifunctional biomaterials that combine electrical conductivity with a flexible, three-dimensional polymeric network.^34^ They typically consist of natural or synthetic polymer matrices with conductive elements integrated into them. Their conductivity stems from electronic conduction, ionic migration, or a synergistic combination of these two mechanisms; these form stable conductive networks within the hydrogel matrix. These networks mimic the endogenous electric field (EF) naturally found after skin injury.^35^ This electrically active microenvironment acts as a bioelectrical signalling platform: it modulates cellular behaviour, guides tissue regeneration, and regulates dynamic changes in the wound microenvironment, which lays the foundation for key conductive mechanisms (*e.g.*, cellular electrotaxis, signalling pathway activation, immune modulation, and integrated antibacterial, drug delivery, and sensing capabilities) that drive the hydrogels' therapeutic effects.^20^

### Cellular electrotaxis

**Fig. 2:**
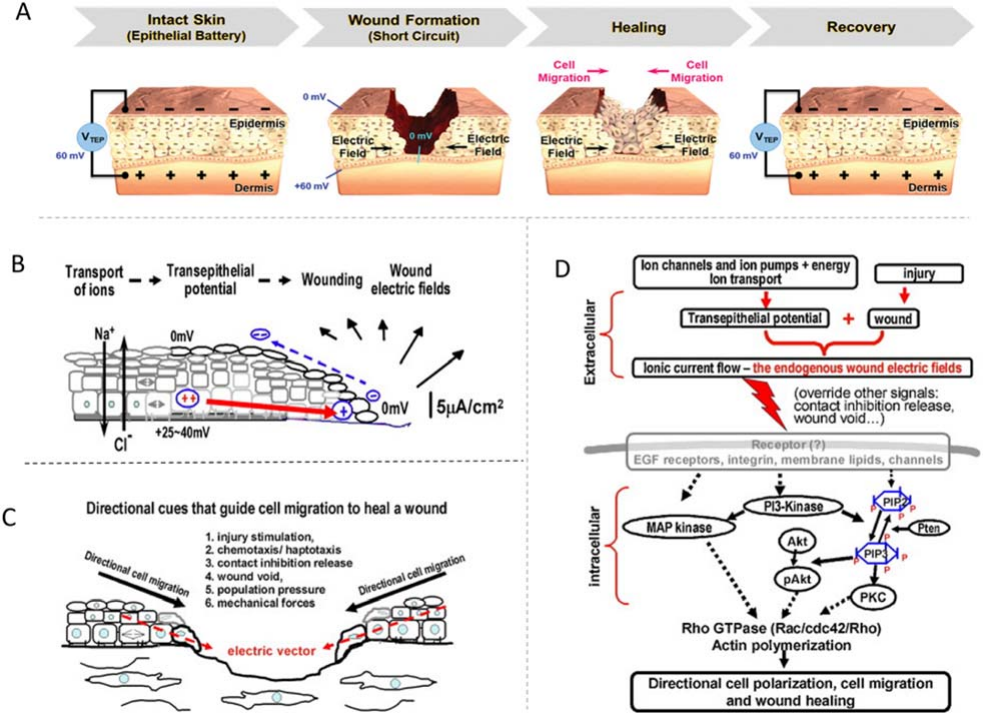
Endogenous wound electric fields. (A) TEP and electric field at the wound site before and after healing. This panel depicts the variation in the electric field throughout wound healing. The figure has been reproduced from ref. 37 with permission from Wiley, copyright 2020. (B) The mechanism of generation of wound electric fields. (C) Directional cues that guide the epithelial cells to migrate directionally at a wound. The steps of cell migration are injury stimulation, chemotaxis, contact inhibition release, presence of wound void, population pressure, and stress/tensileforces. The endogenous wound electric field generates a persistent vector pointing toward the wound centre (red dashed arrow), guiding migrating cells into the wound. (D) Electrical signalling in cell migration and wound healing. The schematic shows the generation of the endogenous wound electric signals and their integration into some critical signalling pathways for cell polarisation, migration, and wound healing. These figures have been reproduced from ref. 20 with permission from Elsevier, copyright 2009.

The electrical conductivity of human skin typically ranges from 2.6 to 1 × 10^−4^ mS cm^−1^.^36^ Epithelial tissue in the epidermis is responsible for ion transport and forming a *trans*-epithelial potential (TEP), this potential usually falls between 10 and 60 mV (Fig. 2A).^37^ When the skin is injured, a short-circuit of current occurs at the wound site, causing the local potential to drop and become negative relative to the undamaged epidermis that lies further from the wound. As a result, current flows toward the wound and creates a transverse wound electric field. Positive charges then move from the surrounding tissues toward the wound and exit through the wound site (Fig. 2B).^20^ Endogenous electrical stimulation (ES) may guide cells to migrate and proliferate along this electric gradient. This process continues until the wound heals and the original TEP is restored.^37^ The current induced by the wound can stimulate tissue growth, and this phenomenon is known as electrotaxis or electrokinetic migration (Fig. 2C).

When electrodes are placed at the wound site and an external current is applied, researchers can replicate the endogenous electric field inside the wound. This replication helps speed up the healing process. However, using external electronic devices often requires complex and time-consuming procedures. These procedures can be inconvenient for both patients and clinicians.^38^ Conductive dressings provide a practical alternative: they adjust the wound's TEP by forming a closure current and promote cell migration while enhancing wound healing.^34^

### Signalling pathway activation

Conductive hydrogel dressings mimic endogenous bioelectric signals and promote cell proliferation and migration by activating relevant cellular signalling pathways. When combined with electrical stimulation (ES), they regulate epithelial cell proliferation and migration through pathways such as MAPK-ERK1/2 and PI3K/Akt (Fig. 2D).^20,39^ ES also promotes angiogenesis and fibroblast growth *via* multiple mechanisms, essential for tissue repair and regeneration.

For angiogenesis, studies have shown that ES stimulates vascular endothelial cells and mesenchymal stem cells (MSCs) to release pro-angiogenic factors, including vascular endothelial growth factor (VEGF) and fibroblast growth factor 2 (FGF2).^40–42^ In addition, ES directly activates the VEGF receptor (VEGFR) signalling pathway in endothelial cells. This activation promotes cell polarisation and directional migration, which contributes to the formation of lumen-like structures.^43^ ES further enhances endothelial cell proliferation and migration by activating the MAPK/ERK signalling pathway.^40^ Konstantinou *et al.* demonstrated that microcurrent stimulation can activate the MAPK signalling pathway and promote the release of TGF-β1.^44^ Another mechanism involves ES enhancing angiogenic capacity by promoting the release of MSC-derived exosomes; these exosomes then indirectly activate the PI3K/Akt and ERK1/2 signalling pathways.^45^ Mohana Sundaram *et al.*^46^reported that electrical stimulation increases the permeability of blood vessel walls. This effect helps transport white blood cells and oxygen to wound sites, which in turn accelerates wound repair.

Regarding fibroblasts, ES enhances cellular activity not only by regulating the cell cycle and elevating intracellular Ca^2+^ levels, but also by activating downstream Ca^2+^/calmodulin-dependent signalling cascades that drive proliferation and migration.^47,48^ ES has further been shown to accelerate myofibroblast transdifferentiation through the TGF-β1/Smad pathway and to stimulate collagen synthesis *via* PI3K/Akt and MAPK/ERK signalling, thereby facilitating ECM remodelling and wound contraction.^49^ In diabetic wounds, low-intensity ES significantly increases fibroblast motility and survival, partly through the activation of ERK1/2 and focal adhesion kinase (FAK) pathways, which restore impaired migratory capacity.^50^

Overall, conductive hydrogels used in combination with ES systematically promote angiogenesis and fibroblast proliferation by activating key signalling pathways. This coordinated action ultimately accelerates tissue repair and regeneration.

### Immune modulation

In addition, conductive hydrogels further support tissue regeneration by modulating the polarisation state of immune cells, particularly macrophages. Macrophages can be broadly divided into two phenotypes: M1, which are pro-inflammatory and primarily involved in the early immune response; M2, which have anti-inflammatory properties and help facilitate tissue repair and angiogenesis.^51^ Studies have shown that direct current ES increases the proportion of M2 macrophages in regenerating tissues and upregulates key M2 marker genes, including interleukin-10 (IL-10) (an anti-inflammatory cytokine that suppresses excessive immune responses), CD163 (a scavenger receptor specifically expressed on M2 macrophages to mediate hemoglobin metabolism), and peroxisome proliferator-activated receptor gamma (PPARG) (a transcription factor that drives M2 polarization and lipid metabolism).^52^ Furthermore, ES reduces CD86 (a marker associated with M1 polarisation) surface expression and inhibits the secretion of pro-inflammatory cytokines, including interleukin-1β (IL-1β) and interleukin-6 (IL-6), thereby promoting tissue repair and functional regeneration.^53,54^

### Antibacterial, drug delivery, and sensing

Moreover, several components commonly used in conductive hydrogels, such as silver nanoparticles (AgNPs),^55^ reduced graphene oxide (rGO),^56^ and polypyrrole (PPy),^57^ possess intrinsic antibacterial and antioxidant properties. These components work synergistically to scavenge reactive oxygen species (ROS), inhibit bacterial adhesion and proliferation, and improve microenvironmental homeostasis in chronic wounds.

Beyond their intrinsic bioactivity, the 3D network structure of conductive hydrogels allows them to act as controlled drug delivery platforms. When exposed to external stimuli like electrical signals or pH changes, these hydrogels can enable precise spatiotemporal drug release through network contraction or swelling, as well as electrostatic repulsion.^58,59^ This mechanism is widely used for the localised delivery of anti-inflammatory agents, growth factors, and antimicrobial peptides. This approach extends the activity of locally delivered drugs while minimising systemic side effects.^60,61^

Additionally, conductive hydrogels have excellent resistive and impedance–responsive properties. These characteristics make them well-suited for use as wound sensors, enabling real-time monitoring of parameters such as pH, humidity, conductivity, and glucose levels. This monitoring capability supports their integration into innovative dressing systems.^35,39^

In summary, conductive hydrogels create an electrically active microenvironment that allows regulation through multiple cellular, tissue, and molecular pathways. They support a range of functions, including promoting regeneration, fighting infection, enabling controlled drug release, and providing intelligent monitoring. These capabilities make them a promising class of high-performance functional dressings. The following section will further explore advances in the application of conductive hydrogel dressings from the perspective of functional categories.

### Common conductive materials

**Fig. 3:**
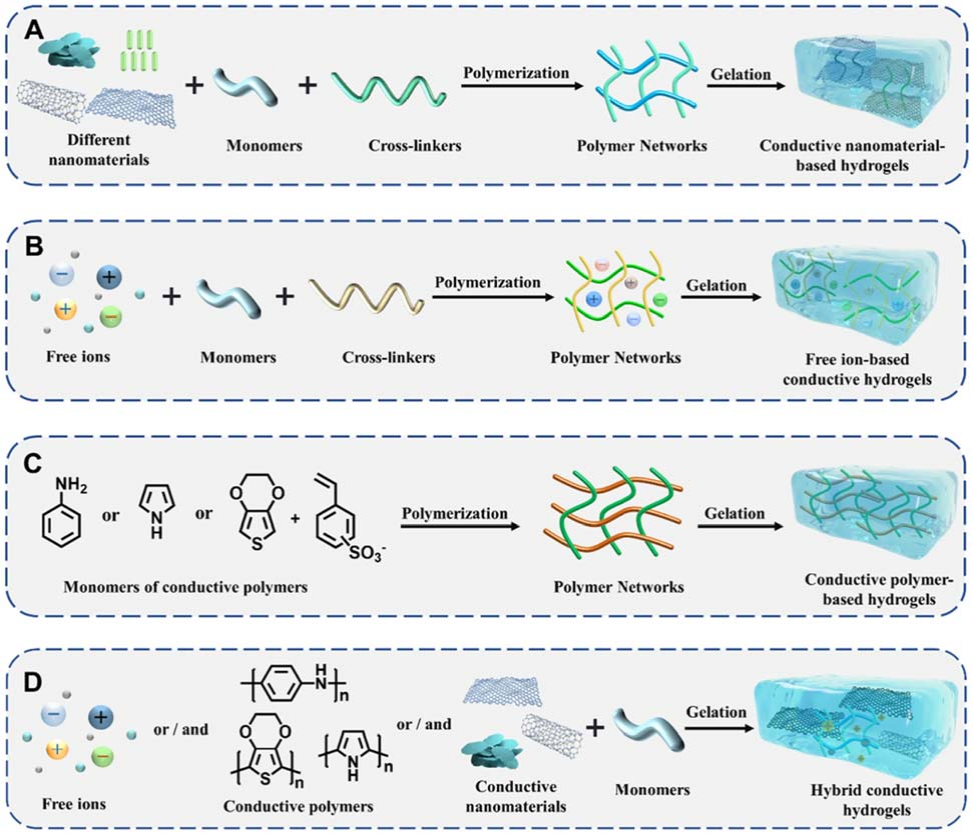
Schematic of the fabrication of conductive hydrogels incorporating different types of conductive fillers: (A) conductive nanomaterials, (B) free ions, (C) conductive polymers, and (D) hybrid conductive systems. This figure was reproduced from ref. 68 with permission from Springer Nature, copyright 2024.

Hydrogels consist of 3D network structures formed by cross-linked natural or synthetic macromolecules. These structures endow hydrogels with excellent biocompatibility, tunable mechanical properties, and strong adherence to tissue surfaces.^62^ The most commonly used natural bioactive polymers include chitosan (CS),^63,64^ hyaluronic acid (HA),^65^ and gelatin,^66,67^ all of which exhibit excellent biocompatibility and biodegradability. However, conventional hydrogels typically have poor electrical conductivity.^68^ To address this limitation, researchers have developed electroactive composite hydrogel networks by incorporating conductive materials. This modification allows the hydrogels to respond to electrical stimuli and facilitate signal transmission in applications such as tissue engineering and wound repair (Fig. 3).^25,69^ A wide range of conductive materials has been used, owing to their distinct conductive mechanisms and broad applicability. Table 1 provides a summary of conductive materials used in hydrogel wound dressings.

**Table 1:** Summary of conductive biomaterials

Type	Component	Composite strategies	Material features	Applications	Ref.
Carbon-based materials	Carbon nanotubes (CNTs)	Carboxylation, PEG grafting, physical doping, self-assembly	High conductivity, enhanced mechanical properties but prone to aggregation	Improved cell migration, mechanical reinforcement, electrical stimulation responsiveness	115
	Graphene/graphene oxide (GO/rGO)	Hydrogen bonding/covalent crosslinking, dopamine coating, electrostatic interactions	Abundant functional groups, good dispersibility, antibacterial/antioxidant activity	Antibacterial conductive dressings, chronic wound repair, controlled drug release	116
	Carbon quantum dots (CQDs)	Carboxyl modification, nitrogen doping, covalent grafting	Strong fluorescence, antioxidant, suitable for smart tracing and ROS regulation	Fluorescence monitoring, ROS regulation, self-healing wound materials	117
	Activated/porous carbon	Simple mixing, physical embedding	Low cost, high surface area, basic conductivity enhancement	Cost-sensitive dressings, auxiliary conductivity improvement	118
Conducting polymers (CPS)	Polyaniline (PANI)	*In situ* oxidative polymerisation, amide/Schiff base grafting	High conductivity, low cost, structural diversity, flexible doping	Chronic wound dressings, burn wound care, electrical stimulation-assisted repair	119
	Polypyrrole (PPy)	*In situ* polymerisation, electrostatic complexation, dopamine coating	High electrochemical stability, good biocompatibility, photothermal sterilisation capability	Self-healing antibacterial dressings, light-responsive antimicrobial hydrogels, deep wound therapy	97
	PEDOT:PSS	Solution doping, photocrosslinking, Ca^2+^ secondary crosslinking, bioprinting	Excellent processability, good flexibility, high biostability, 3D printing compatibility	Smart sensing skins, hydrogel electrodes, bioelectronic interfaces, long-term dressings	120
	Polythiophene (PTh)	Photosensitive doping, self-assembly, nanocomposites	Strong photo-responsiveness, ROS-inducing antibacterial activity, stable conductivity	Photoelectric anti-infection dressings, photothermal/electroactive combination therapy	121
Metals/metal oxides	Silver nanoparticles (AgNPs)	*In situ* reduction (*e.g.*, ascorbic acid), physical doping, ionic complexation	Extremely high conductivity, broad-spectrum antibacterial activity, tunable size	Antibacterial conductive dressings, burn/infected wound repair, accelerated chronic wound closure	122,123
	Zinc/zinc oxide (Zn^2+^/ZnO)	Ion coordination crosslinking, nanoparticle embedding, stimuli-responsive composites	Promotes cell proliferation and angiogenesis, anti-inflammatory, low cost and non-toxic	Chronic wounds, diabetic wound healing, skin regeneration materials	124
	Gold nanoparticles/nanowires (AuNPs/AuNWs)	Physical embedding, surface grafting, hydrophobic-hydrophilic self-assembly	Excellent stability, good biocompatibility, safe for long-term implantation	Bioelectronic interfaces, skin electrodes, neural dressings, precision repair platforms	125
	Copper nanoparticles (CuNPs)	Physical doping, chelation/complexation reactions, core–shell coating (*e.g.*, dopamine)	Conductivity plus angiogenesis promotion, strong antibacterial activity	Ischaemic wounds, vascular-stimulating dressings	126

### Carbon-based materials

Carbon-based materials are considered ideal conductive components for developing conductive hydrogels owing to their excellent electrical conductivity, chemical stability, biocompatibility, and cost-effective, scalable synthesis.^70^ Common carbon materials include carbon nanotubes (CNTs), graphene, graphene oxide (GO), reduced graphene oxide (rGO), activated carbon, carbon fibres, carbon quantum dots, and mesoporous carbon.^71^

CNTs form effective electrical pathways within hydrogel networks, enabling the transmission of bioelectric signals at wound sites. This enhances stable interactions between cells and the ECM, which in turn guides cell migration, supports angiogenesis, and ultimately accelerates tissue repair and regeneration.^72^ GO has high surface electronegativity, which can promote the condensation of cationic polymers through electrostatic interactions, boosting the bioactivity of graphene-based materials hydrogels.^73^ The antibacterial properties of GO stem from its sharp edges, which can induce oxidative stress on bacterial membranes.^74,75^ Beyond their antibacterial activity, GO and rGO have excellent electron-transport capabilities, allowing them to transmit endogenous bioelectric signals and participate in cellular electrical activities. These features make GO-based hydrogels particularly suitable for wound healing applications that require antimicrobial protection and electrical stimulation to promote tissue regeneration.

**Fig. 4:**
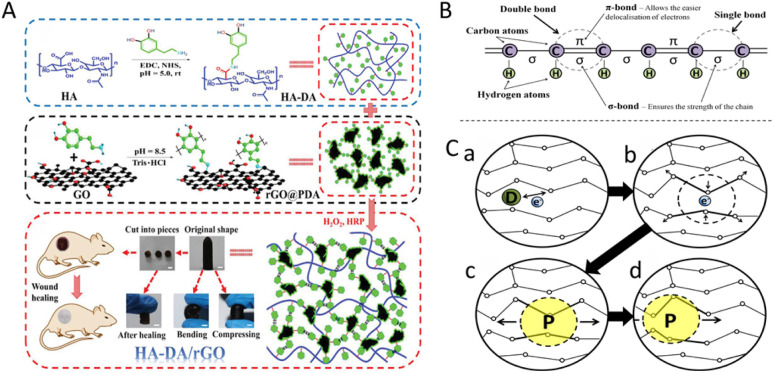
(A) Reduced graphene oxide (rGO) is incorporated into hydrogel formulations to serve as a wound-dressing material. This figure was reproduced from ref. 77 with permission from small, copyright 2019. (B) Simplified schematic of a conjugated backbone: a chain containing alternating single and double bonds. (C) A simplified explanation of the electrical conductivity of conducting polymers. (a) The dopant adds or removes an electron, generating a delocalised charge on the polymer chain. (b) This charge tends to localise with an accompanying lattice distortion. (c) Such a charge–distortion complex is called a polaron. (d) Polarons can move along the polymer chain, enabling electrical conduction. These figures were reproduced from ref. 97 with permission from Elsevier, copyright 2014.

rGO exhibits good biocompatibility and adhesion properties, and acts as an innovative antibacterial agent due to its strong antimicrobial efficacy.^76^ Additionally, rGO helps form conductive hydrogel networks that enable bioelectronic communication at the wound site. These carbon nanomaterials can be incorporated into hydrogel matrices (*e.g.*, chitosan, hyaluronic acid, and gelatin) through simple physical blending or *in situ* gelation methods. This allows the construction of electrically active network structures without the need for complex procedures (Fig. 4A).^77^ However, carbon materials are inherently prone to agglomeration and hydrophobicity. To overcome these limitations, researchers have developed various surface functionalization strategies that improve the dispersion, colloidal stability, and biosafety within the hydrogel matrix.^78,79^

### Conductive polymers (CPs)

Conductive polymers (CPs) are a class of polymers that exhibit intrinsic electrical conductivity. CPs include polyaniline (PANI), phenylamine oligomers, polypyrrole (PPy), poly(3,4-ethylenedioxythiophene):poly(styrene sulfonate) (PEDOT:PSS), and polythiophene (PTh). These polymers have π-conjugated structures that enable electron conduction and ionic migration through alternating single and double bonds. This structure supports efficient charge transport within hydrogel networks (Fig. 4B).^60^ The conductivity of these polymers ultimately depends on the dopants. These polymers are synthesised in an oxidised, conductive form, and dopant anions are essential for stabilising the polymer backbone and maintaining charge neutrality (Fig. 4C).^80^ This conductive functionality promotes cell proliferation, migration, adhesion, and differentiation, thereby supporting the wound healing process.^37,81^ However, most CPs are inherently hydrophobic, poorly soluble in aqueous media, and have limited biocompatibility. Therefore, chemical and/or physical modifications are often needed to improve their mechanical strength, biocompatibility, electrical conductivity, and solubility.

PANI is synthesised through the 1,4-coupling reaction of aniline monomers, followed by oxidative polymerisation in the presence of a protonic acid.^82^ However, its conductivity decreases significantly under neutral or alkaline conditions, as dopants leach from the polymer matrix.^83,84^ To overcome this limitation, PANI is often combined with conductive nanomaterials such as graphene, GO,^85^ CNTs,^84^ or metal oxides.^86,87^ This combination greatly enhances the electrochemical stability and conductivity of PANI in physiological conditions. Despite these inherent limits, PANI remains a promising conductive polymer because of its ease of chemical modification, structural adaptability, and ability to form multifunctional composites for hydrogel engineering. Additionally, doping can confer antimicrobial properties to PANI, helping in inhibiting the growth of bacteria and other microorganisms. Thus, although unmodified PANI is not suitable for direct use, its modified or composite forms remain highly in physiological environments attractive options for functional conductive hydrogels. Considering the poor compatibility of PANI in physiological environments, aniline oligomers, especially aniline tetramers (AT), are an attractive option for addressing some of these limitations. Aniline tetramers can be considered a polyaniline chain fragment; they are structurally similar to polyaniline but have a lower molecular weight.^88^ Phenylamine oligomers, owing to the presence of quinone rings, are capable of scavenging reactive oxygen species (ROS), neutralising them and preventing ROS-induced cytotoxicity.^89^

PPy, the most widely used conductive polymer, is commonly synthesised through chemical oxidation (using free-radical initiators in electrolytic media) or electrochemical polymerisation on platinum-coated electrodes.^90^ Due to its excellent electrical properties and stable photothermal behaviour, PPy has been widely used in various wound types, especially infected wounds.^91^ However, its potential cytotoxicity means it requires cautious application.

Among CPs, PEDOT:PSS is considered one of the most successful. It offers high conductivity, injectability, self-healing capability, and mechanical flexibility. PEDOT itself is a conductive polymer with excellent conductivity owing to its π–π conjugated structure, while PSS acts as a polyanionic dopant and dispersant, improving the solubility, processability, and film-forming ability of the composite.^92^ In hydrogel systems, PEDOT:PSS not only enables efficient electrical signal transmission but also contributes to the mechanical tunability and stability. These properties support cell-material interactions and promote tissue regeneration. In animal experiments, applying PEDOT:PSS significantly increased the expression of transforming growth factor-β1 (TGF-β1), confirming the proven wound-healing activity of PEDOT:PSS.^93^ Given its unique properties, this conductive polymer also has broad applications in biomedical sensors, particularly for cardiovascular disease diagnostics.^94^ These attributes give it great potential for biomedical applications.^95^

PTh has attracted considerable attention owing to its aromatic ring structure, which confers good environmental stability, easy preparation, high conductivity, and luminescent properties after doping. Thiophene has an electron-rich aromatic ring that can be oxidised to form polymer films with strong adhesion and high conductivity. It can be electrochemically synthesised as highly adherent films or thick powdery deposits. The uniformity of these films is high but decreases as the film thickness increases.^96^

### Metallic conductive materials

Metallic conductive materials, including silver (Ag), gold (Au), copper (Cu), and zinc (Zn) in the form of nanoparticles (NPs) or nanowires (NWs), are widely used for fabricating conductive hydrogels. Their popularity stems from their excellent electrical conductivity, antimicrobial properties, and tunable nanoscale dimensions.^90,98–100^ In wound-dressing applications, conductive hydrogels incorporating metal ions and their oxides offer dual functionality in wound management: facilitating tissue regeneration through electrical stimulation and preventing infection through antimicrobial activity.^101–103^

Among metallic materials, silver is widely employed for the antimicrobial treatment of burns and infected wounds. Ag ions can form nanoparticles *in situ* in the presence of reducing agents and can be readily incorporated into hydrogels, thereby broadening their range of applications.^104^ Au is another widely used metal in conductive biomaterials, with Au nanoparticles and nanorods used to design conductive hydrogels.^105,106^ Under visible-light irradiation, gold nanoparticles can generate photocatalytic ROS, which exert antibacterial effects and further support tissue repair.^107^ However, metal nanomaterials tend to undergo oxidative degradation and aggregation during use. To overcome these limitations, researchers use surface modification strategies, such as coatings based on polyethylene glycol (PEG, a biocompatible polymer commonly used to improve stability and reduce protein adsorption) or dopamine, to enable stable encapsulation and controlled release.^108^ Nonetheless, ion release may damage cellular membranes and mitochondrial function, potentially causing cytotoxic effects.^33^ Thus, balancing therapeutic efficacy and biosafety remains a key challenge for the biomedical application of metal-based conductive hydrogels.

### Other materials

MXenes (Ti_3_C_2_T_*x*_), two-dimensional materials consisting of transition metal carbides and carbonates, are characterised by exceptional biocompatibility, advantageous surface hydrophilicity, a capacity for surface functionalization, high electrical conductivity, and remarkable mechanical properties.^109,110^ In MXene-impregnated hydrogels, the nanosheets undergo stable interactions with the hydrogel matrix *via* their surface functional groups. This not only enhances the electrical conductivity of the hydrogel but also preserves its flexibility and tunable mechanical properties.^60^ This material has shown promising effects in promoting wound healing but is still in the early stages of clinical translation for wound-care applications.^111,112^

Another two-dimensional conductive material, black phosphorus (BP), is a layered two-dimensional semiconductor with distinctive electrical and optical characteristics.^113^ Researchers have also incorporated significant amounts of free ions into hydrogels, imparting excellent ionic conductivity, rapid gelation, injectability, and high elasticity. Ionic conductive hydrogels can better mimic ion transport in tissues and cells, creating a sustained ionic conductive microenvironment for skin wounds and supporting cell migration and proliferation.^60,114^

Furthermore, future research is expected to focus on the rational design of hybrid hydrogel systems that integrate multiple conductive components, such as MXenes, BP, CPs, and ionic fillers, to achieve synergistic effects. These multifunctional hydrogels could provide enhanced electrical responsiveness, controlled drug delivery, antibacterial activity, and real-time sensing capabilities. In turn, this would help meet the complex demands of advanced wound management.

## Conductive hydrogel for diverse wounds

Wound dressings play a crucial role in wound care and cutaneous tissue regeneration.^127^ To meet the needs of different wound types, various commercial products have been developed. Choosing appropriate wound dressings is essential for promoting wound healing. Conductive hydrogels, with their superior conductivity, flexibility, biocompatibility, and inherent antimicrobial activity, can mimic the electrophysiological microenvironment of human tissue. This ability supports cell adhesion, proliferation, and migration, which is why they have attracted significant attention in the biomedical field, especially for wound repair.^128^ They also have tunable mechanical properties and strong formability, allowing them to conform to various irregular wound surfaces. This feature highlights their unique advantages in innovative dressing systems.^129^ Conductive hydrogels are used to treat burns, infections, chronic diabetic wounds, and joint wounds. Table 2 provides a summary of conductive hydrogel dressings for different types of wounds.

**Table 2:** Summary of conductive hydrogel dressings for diverse wounds

Application	Name of dressing	Composition	Main features	Conductivity(S m^−1^)	Ref.
Burn wounds	TG/SF^Van^ and CAP@DA	Silk fibroin (SF), gum tragacanth (TG), carboxyl-capped aniline pentamer (CAP), dopamine (DA)	Drug delivery, antibacterial, biocompatibility, conductivity, antioxidant properties, mechanical strength	3.42 × 10^−6^	133
	HPD_ChCl_ gel	Choline chloride (ChCl), betaine (Bet), glycerol (Gly), deep eutectic solvents (DESs), hyaluronic acid (HA)	Ion-channel conductivity, stability, mechanical strength, self-healing, adhesion, antibacterial capability	0.25 ± 0.05	134
	GelDA/pGO hydrogel	Dopamine-grafted gelatin (GelDA), polydopamine-coated graphene oxide (pGO)	Adhesion, hemostatic performance, conductivity, antioxidant ability, mupirocin drug release, photothermal antibacterial, biocompatibility	7.2 × 10^−2^	150
	PVA/OPGFs hydrogel	Oxygen plasma-treated graphene fibres (OPGFs), poly(vinyl alcohol) (PVA)	Conductivity, resistance-strain sensitivity, cooling and healing efficacy, biocompatibility, excellent mechanical properties	101 ± 0.75× 10^−3^	151
	SF/CMC/AG and GO@PDA hydrogel	Graphene oxide coated with polydopamine (GO@PDA), silk fibroin (SF), carboxymethyl cellulose (CMC), agarose	Vancomycin release, drug delivery, antibacterial, biocompatibility, injectable, mechanical and electrical conductivity, antioxidant properties	5.6 × 10^−3^	152
Infected wounds	OSD/CMC/Fe/PA hydrogel	Sodium alginate (SA), oxidised sodium alginate (OSA), dopamine (DA), carboxymethyl chitosan (CMC), poly(thiophene-3-acetic acid) (PTAA), Fe^3+^	Conductivity, photothermal antibacterial activity, tunable rheology, suitable mechanical strength, antioxidant properties, tissue adhesion, hemostasis	3.43 × 10^−4^	31
	QCSG/GM/GO hydrogel	Glycidyl methacrylate-functionalised quaternised chitosan (QCSG), gelatin methacrylate (GM), graphene oxide (GO)	Photothermal conductivity, antibacterial, biocompatibility, biodegradability, antibiotic release	10.07 ± 2.69 × 10^−2^	153
	GT-AT*x*/QCS/CD hydrogel	Gelatin (GT), aniline tetramer (AT*x*), quaternised chitosan (QCS), β-cyclodextrin (β-CD)	Flexibility, tissue adhesion, self-healing, injectability, biocompatibility, antioxidant, conductivity, intrinsic and photothermal antibacterial activity	0.4–0.8 × 10^−3^	154
	Ag NPs/CPH hydrogel	Polyvinyl alcohol (PVA), gelatin, silver nanoparticles (Ag NPs), phytic acid (PA)	Mechanical strength, porous structure, good electrical conductivity, effective antibacterial properties, low toxicity, healing ability	0.069	138
	CEC/PF/CNT hydrogel	*N*-carboxyethyl chitosan (CEC), benzaldehyde-terminated PF127 (PF127-CHO), carbon nanotubes (CNTs)	pH-responsive moxifloxacin release, antibacterial, photothermal antibacterial activity, adhesive, mechanical properties, conductivity, self-healing	1.37 × 10^−3^	155
	H-L and H-NP hydrogel	Lignin, phenylboronic acid-modified hydroxypropyl cellulose (PAHC), 4-carboxyphenylboronic acid	Hemostatic, antibacterial, antioxidant, conductive properties	0.085	156
	COGFe hydrogel	Carboxymethyl chitosan (CMC), oxidised sodium alginate (OSA), gallic acid (GA), Fe^3+^	Toughness, conductivity, adhesion, self-healing, antimicrobial activity, photothermal antimicrobial capacity	1 × 10^−5^–0.26	157
	GT-DA/CS/CNT hydrogel	Chitosan (CS), gelatin-grafted dopamine (GT-DA), carbon nanotubes (CNTs)	Doxycycline release, photothermal antibacterial activity, conductivity, antioxidant properties, mechanical strength, shape recovery	2.5 × 10^−2^	72
	PSMT hydrogel	Ti_3_AlC_2_ (MAX phase), polyvinyl alcohol (PVA), europium (Eu), tannic acid (TA)	Antioxidant, electrical conductivity, biocompatibility, antibacterial, photothermal properties	0.1–0.15	158
	PVA/POA-MX@Mg^2+^ hydrogel	Polyvinyl alcohol (PVA), metal carbon/nitride (MXene), Mg^2+^, oxidised hyaluronic acid (POA)	High antibacterial efficiency, suitable conductivity, excellent self-healing properties, favourable biocompatibility	—	139
	HA-CYS/PFA/PDA@PPy hydrogel	Hyaluronic acid (HA), polydopamine@polypyrrole nanocomposite (PDA@PPy), cystamine (CYS), poly(ethylene glycol)-*co*-poly(glyceryl sebacate) (PFA)	UV-blocking ability, self-healing, injectability, tissue adhesion, photothermal anti-infection capability	0.97	159
	CP/OD hydrogel	Oxidised dextran (OD), chitosan-graft-polyaniline (CP)	pH-responsive amoxicillin loading and release, injectability, biocompatibility, biodegradability, conductivity	7.9 × 10^−2^	160
Diabetic wounds	PVA-CEC-AGA/Ag hydrogel	Polyvinyl alcohol (PVA), carboxyethyl chitosan (CEC), agarose, silver (Ag)	On-demand dissolvability, stimuli-responsive behaviour, mechanical self-healing, transparency, antibacterial ability, conductivity	—	32
	PACPH hydrogel	Polydopamine (PDA), cellulose nanocrystals (CNC), silver nanoparticles (Ag NPs), polypyrrole (PPy)	Mechanical strength, antibacterial, photothermal performance, tissue adhesion, electroactivity	—	161
	PDA@Ag NPs/CPHs	Polydopamine-decorated silver nanoparticles (PDA@Ag NPs), polyaniline, polyvinyl alcohol (PVA)	Sensing, tunable mechanical properties, antibacterial, electrochemical performance, repeatable adhesiveness	—	162
	PCPZ hydrogel	Polypyrrole (PPy), polyvinyl alcohol (PVA), chitosan, zinc (Zn)	Antibacterial, self-healing, temperature and strain sensing, conductivity, mechanical properties	1.16	163
	PIL-OHA hydrogels	Oxidised hyaluronic acid (OHA), *N*-(3-aminopropyl)imidazole, 1,2-dibromoethylene, ammonium persulfate (APS), sodium periodate	Electrical conductivity, flexibility, mechanical properties, antibacterial activity	0.76 × 10^−3^	164
	QP-P-D hydrogel	Polyaniline (PANI), four-armed aldehyde-terminated polyethylene glycol (4-arm PEG-CHO), chitosan, deferoxamine (DFO)	Antibacterial, self-healing, conductive, injectable	—	165
	PPCA hydrogel	Silver nanoparticles, polypyrrole (PPy), cobalt ions (Co^2+^), poly(acrylic acid) (PAA), branched poly(ethylenimine) (PEI)	Conductivity, mechanical strength, antibacterial activity, cytocompatibility	0.048	166
	PQCD-A@Cur hydrogel	Curcumin (Cur), artificial all-melanin nanoparticles (AMNPs), polyaniline-grafted quaternised chitosan (PQCS)	NIR-responsive curcumin release, antioxidant, anti-inflammatory, neurotrophic, self-healing, mechanical strength, photothermal properties	4.1 × 10^−3^	167
	HA-PA-rhAM hydrogel	Hyaluronic acid (HA), phytic acid (PA), recombinant human amelogenin (rhAM)	Mechanical strength, stability, electrical conductivity, adaptation to irregular wound shapes	1.75 × 10^−3^	168
	G-Ppy and gel-MA/Chi-C/G-Ppy hydrogel	Histatin-1 (His-1), polypyrrole-based conductive nanoparticles (G-Ppy), methacryloyl-grafted gelatin (Gel-MA)	Good adhesion, stability, biocompatibility, mechanical properties, conductivity, anti-inflammatory activity	4.44 × 10^−4^	169
	PEG/Ag/CNT-M + E hydrogel	Multiwalled carbon nanotubes, angiogenesis, four-armed SH-PEG	Tissue adhesiveness, antioxidant properties, self-healing, electrical conductivity, metformin loading	3.04–3.65 × 10^−4^	140
Joint wound	PSPAg hydrogel	Ag nanoparticles(AgNPs), silk fibroin (SF), polyaniline (PANI), poly(AM-*co*-SBMA)	Mechanical properties, excellent self-adhesive performance, exceptional sensing capability, superb antibacterial performance, conductive and fantastic biocompatibility	1.87–8.38 × 10^−2^	170
	Has	Hydroxypropyl trimethylammonium chitosan chloride (HACC) and sodium alginate (SA)	Conductivity, biocompatibility, high flexibility and mouldability, wound monitoring	1.14	149
	Alg-PBA/PVA/GOH hydrogel	Aminophenylboronic acid-grafted sodium alginate (Alg-PBA), polyvinyl alcohol (PVA), hydroxylated graphene (GOH)	Rapid self-healing (within 60 s), injectable, conductive, motion monitoring, antibacterial	2.26 × 10^−3^	148
	PEGSD-Zn^2+^/PHA-I hydrogel	Poly(glycerol sebacate)-*co*-poly(ethylene glycol)-g-catechol(PEGSD)/Zn^2+^/(3-acrylamidophenyl) boronic acid and 2-hydroxyethyl acrylate/ionic liquids	Flexibility, tissue adhesiveness, antibacterial, antioxidant, conductive, biocompatibility, sensing capability	5 × 10^−4^ -1.8 × 10^−3^	171

### Burn wounds

Burns are a common form of tissue injury resulting from exposure to flames, high temperatures, or radiant thermal energy.^130^ Severe burns often lead to extensive tissue necrosis, damage to blood vessel structures, and prolonged chronic inflammatory responses.^131^ These pathological changes disrupt the skin's *trans*-TEP, weakening the endogenous EF, and impairing the migration and proliferation of electrically responsive cells, including endothelial cells, macrophages, and fibroblasts. Consequently, burn wounds frequently heal slowly, have a higher risk of scarring, and are more susceptible to infection.

**Fig. 5:**
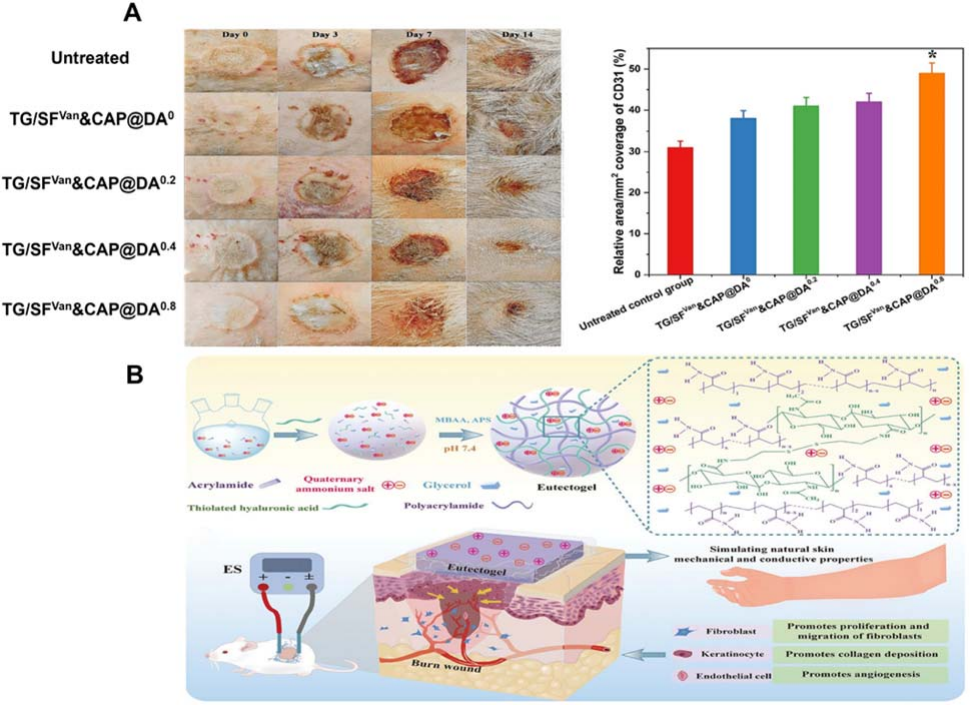
(A) Images depicting burn wounds at different time intervals and the quantitative expression of CD31 (a critical marker for endothelial cell identity and vascular integrity). These figures were reproduced from ref. 133 with permission from Elsevier, copyright 2024. (B) Schematic of the design strategy and application of conductive DN eutectogel. This figure was reproduced from ref. 134 with permission from Wiley, copyright 2024.

Conductive hydrogel dressings support tissue regeneration and accelerate burn-wound healing through antimicrobial action, microenvironmental modulation, and electrical stimulation. Together, these functions make them promising therapeutic agents for advanced burn management.^132^ Babaluei *et al.*^133^ developed a conductive hydrogel using gum tragacanth (TG) and silk fibroin (SF) as the base, with carboxyl-capped aniline pentamers (CAP@DA) added as the conductive component. In a rat model of third-degree burns, the results showed that wound healing in the hydrogel-treated group was significantly better than in both the untreated group and the low-component hydrogel group (Fig. 5A). As the concentration of CAP@DA increases, the conductivity of the hydrogel also increases, similar to human skin. This conductivity influences Ca^2+^ channels, stimulates the expression of pro-angiogenic factors, and promotes angiogenesis. These findings highlight a key design principle: tuning the conductivity of hydrogels can directly affect cellular signalling and tissue regeneration. While this study represents progress in burn wound treatment, it relies primarily on rat models. Given the fundamental differences in physiological structures between rodents and humans, translating these findings to clinical practice may pose challenges. Additionally, potential issues such as long-term biocompatibility and possible immune responses require further investigation.

Although conductive hydrogels have shown significant potential as electroactive dressings for accelerating burn wound healing, they still face several challenges. These include balancing the electrical conductivity with mechanical strength, the tendency to dehydrate, and limited optical transparency. To address these limitations, Tian *et al.*^134^ developed a dual-network conductive eutectic hydrogel. They integrated polyacrylamide/choline chloride/glycerol with thiolated hyaluronic acid and polymerisable deep eutectic solvents (PDESs). The conductivity of the hydrogel comes from the migration of choline chloride within the gel network. This co-gel can match the electrical conductivity of natural human skin (up to 0.25 S m^−1^) and has high tensile strain. It also exhibits strong tissue adhesion, intrinsic self-healing ability, and antimicrobial activity. When used with external electrical stimulation, the conductive co-gel effectively reduces inflammation, stimulates cell proliferation and migration, and supports collagen deposition, neovascularisation, and skin tissue remodelling (Fig. 5B). However, the long-term stability of the electrical conductivity of this hydrogel *in vivo* still needs to be investigated.

### Infected wounds

Bacterial infections are common complications during wound healing and can lead to serious consequences that significantly delay the repair process. Microbial colonisation on wound surfaces triggers inflammatory responses, hinders re-epithelialization, prolongs the healing period, and imposes substantial physical and financial burdens on patients.^135,136^ The widespread use of antibiotics has worsened the growing problem of antimicrobial resistance.^137^ Consequently, developing effective antimicrobial dressings remains a major challenge in bioengineering. In this context, certain conductive materials with intrinsic antimicrobial properties can serve as alternative antibiotics. This reduces overreliance on antimicrobial agents and lowers the risks associated with the overuse of antibiotics. Researchers are actively exploring ways of incorporating antibiotics, cationic polymers, inorganic metal ions (*e.g.*, Ag^+^, Zn^2+^, Cu^2+^), and metal oxides into the structural framework of conductive hydrogels to boost their antimicrobial efficacy.^136,138^

**Fig. 6:**
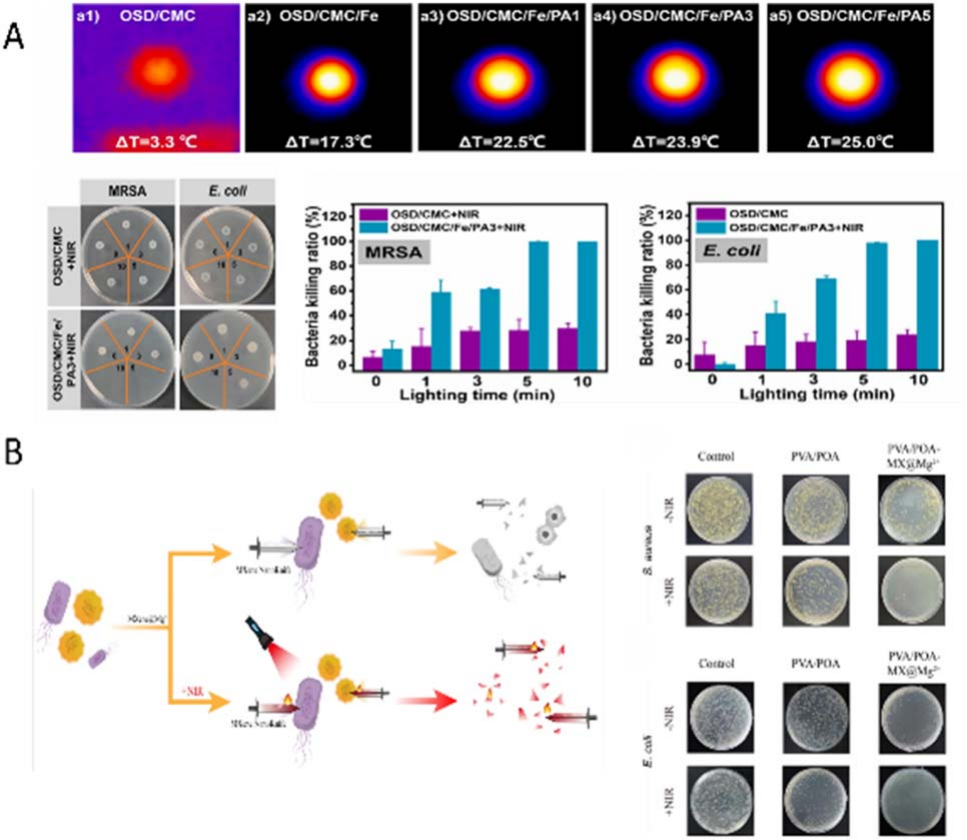
(A) Schematic of the photothermal antibacterial effect of the OSD/CMC/Fe/PA3 hydrogel. These figures were reproduced from ref. 31 with permission from Elsevier, copyright 2023. (B) Antibacterial mechanism and effects of the PVA/POA-MX@Mg^2+^ hydrogel. These figures were reproduced from ref. 139 with permission from Elsevier, copyright 2025.

To develop a multifunctional hydrogel for treating infectious wounds, Qiao *et al.*^31^ designed a composite material made of sodium alginate grafted with dopamine, carboxymethyl chitosan, and Fe^3+^ (OSD/CMC/Fe). This composite was later combined with poly(thiophene-3-acetate) (PA) to form a viscous, self-healing, conductive, and antibacterial hydrogel dressing (OSD/CMC/Fe/PA). In a murine model of a full-thickness skin defect, treatment with the OSD/CMC/Fe/PA_3_ hydrogel under near-infrared (NIR, 700–2500 nm, which penetrates tissue with minimal photodamage) irradiation reduced the wound area to just 12% after 14 days, markedly smaller than that observed in the control group treated with Tegaderm™ film. *Escherichia coli* and methicillin-resistant *Staphylococcus aureus* (MRSA) were used as representative pathogens. Post-treatment analysis showed significant morphological and structural damage to both bacterial strains. The bacterial kill rate was significantly improved in both *in vitro* and *in vivo* tests (Fig. 6A). The strong antibacterial activity of OSD/CMC/Fe/PA_3_ comes from its excellent photothermal conversion ability and the synergistic antimicrobial effects of carboxymethyl chitosan and Fe^3+^.

MXene is a conductive two-dimensional nanomaterial that has attracted widespread interest in recent years for application in functional hydrogels. Its appeal stems from its high specific surface area, layered structure, excellent conductivity, and unique photothermal conversion capabilities. Wang *et al.*^139^ developed a multifunctional conductive hydrogel using polyvinyl alcohol (PVA) and phenylboronic acid (PBA)-grafted oxidised hyaluronic acid (POA) as the polymeric backbone, incorporating magnesium-ion-modified components. They synergistically incorporated magnesium ion-modified MXene nanosheets (MXene@Mg^2+^) into this backbone to create a PVA/POA-MX@Mg^2+^ hydrogel dressing with excellent overall performance. This system's antibacterial mechanism works through two main pathways: first, the sharp edges of the MXene nanosheets physically damage bacterial membranes, cause cytoplasmic leakage, and ultimately lead to bacterial death; second, MXene has excellent NIR absorption capacity, which allows it to quickly convert NIR energy into thermal energy under NIR laser irradiation. This achieves local photothermal sterilisation (photothermal therapy, PTT). The high-temperature environment not only increases the permeability of bacterial membranes but may also cause protein denaturation and DNA damage, further enhancing the antibacterial effect (Fig. 6B). In vitro and *in vivo* experimental results indicate that the PVA/POA-MX@Mg^2+^ hydrogel has better antibacterial performance, cytocompatibility, and wound healing efficacy than the control group, with clear benefits for treating infected wounds.

### Diabetic wounds

Diabetic wounds are a common chronic injury defined by prolonged infection, abnormal angiogenesis, and delayed epithelial regeneration.^140^ These wounds pose a serious threat to human health, often requiring long-term care and potentially leading to severe complication, limb amputation, or even death.^141^ Diabetic neuropathy affects peripheral nerves, reducing patients' ability to sense pressure and external stimuli. This leads to repeated trauma and impaired wound healing, which often results in ulceration. Additionally, bacterial infections and the buildup of ROS caused by hyperglycaemia further slow the healing process.

**Fig. 7:**
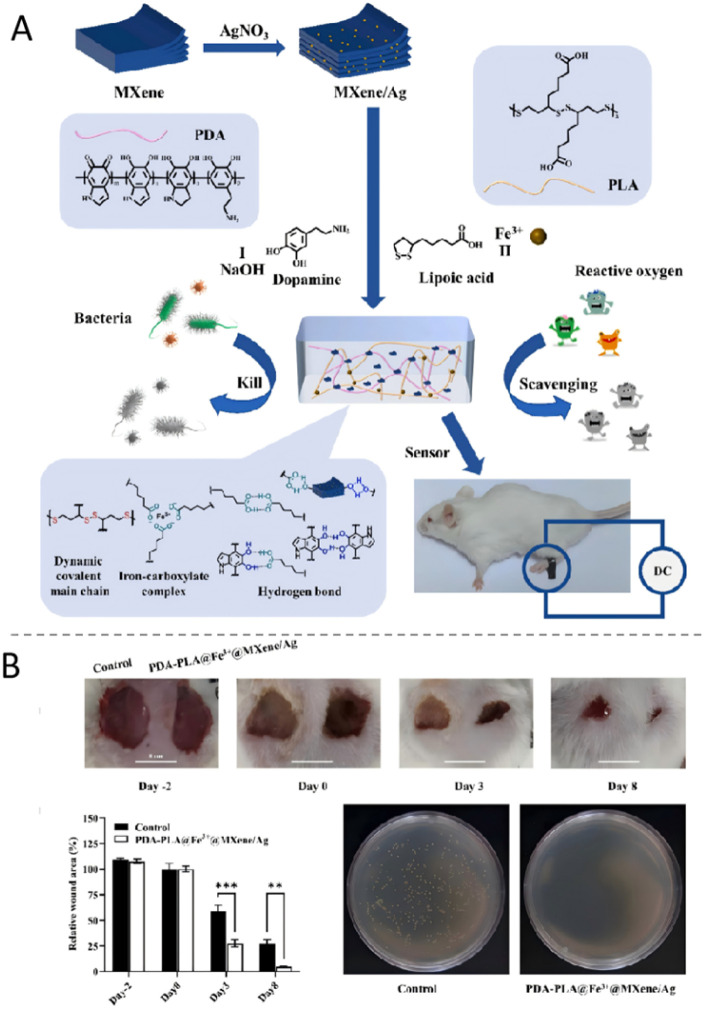
(A) Preparation of the PDA-PLA@Fe^3+^@MXene/Ag hydrogel dressings. (B) Comparison between the PDA-PLA@Fe^3+^@MXene/Ag hydrogel dressings and the control group. These figures were reproduced from ref. 142 with permission from Elsevier, copyright 2025.

To address these challenges, Huang *et al.*^142^ developed a conductive PDA-PLA@Fe^3+^@MXene/Ag hydrogel dressing with epidermal sensing, antimicrobial, and antioxidant functions. The hydrogel combines antibacterial AgNPs with two-dimensional MXene nanosheets (Fig. 7A). These components form a continuous conductive network. When stimulated by ROS, AgNPs are released. Working in synergy with the physical shearing action of MXene, these AgNPs damage bacterial membranes; this accelerates the wound healing process (Fig. 7B). Furthermore, the conductive network converts external stimuli like pressure or heat into detectable electrical signals. This capability enables real-time monitoring of unnoticeable trauma and addresses the sensory deficits linked to diabetic neuropathy.

**Fig. 8:**
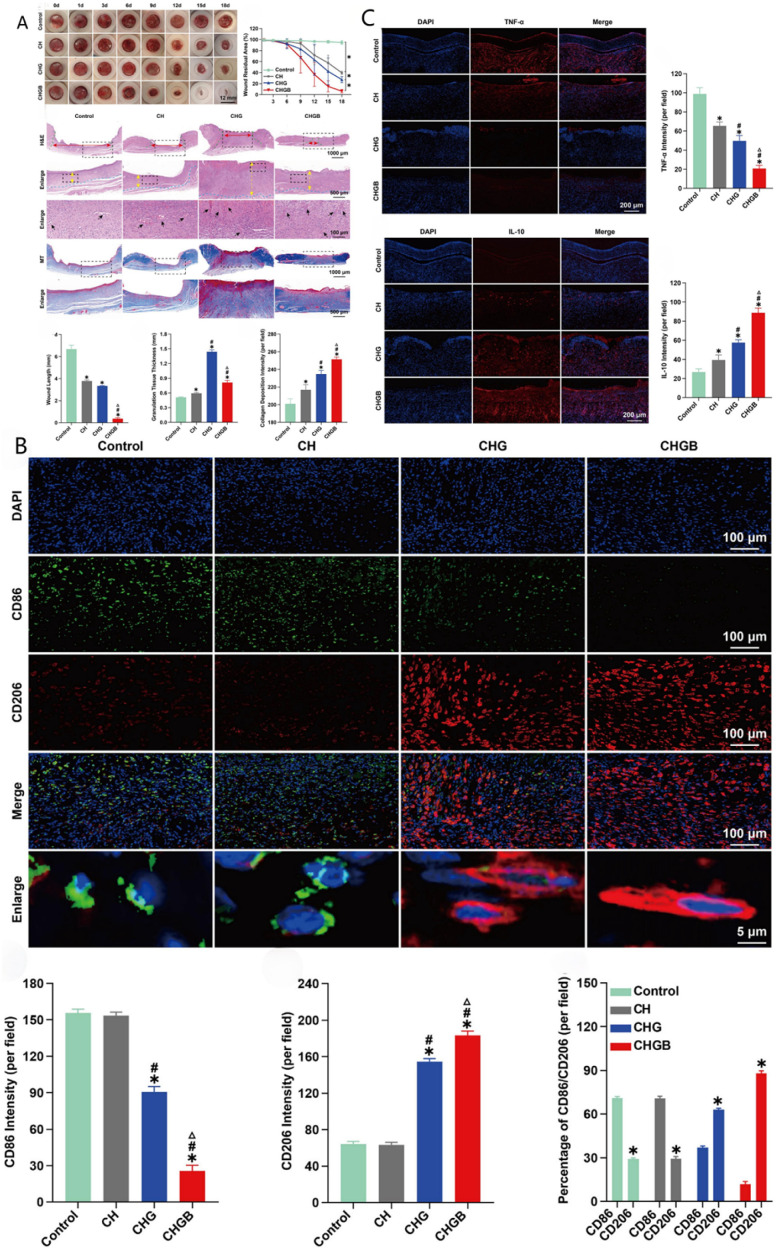
(A) Effects of injectable conductive hydrogel in promoting healing in infected diabetic wounds. (B) Effects of the injectable conductive hydrogel on macrophage polarisation *in vivo*. M1 phenotype macrophages (CD86: green), M2 phenotype macrophages (CD206: red), and nuclei (DAPI, blue). (C) Effects of the injectable conductive hydrogel on inflammation *in vivo*. These figures were reproduced from ref. 144 with permission from Elsevier, copyright 2022.

Immune dysfunction at diabetic wound sites is defined by high levels of pro-inflammatory cytokines (*e.g.*, TNF-α, IL-6) and reduced levels of anti-inflammatory cytokines and growth factors (*e.g.*, IL-10, TGF-β, VEGF). This creates a chronic inflammatory microenvironment that slows wound healing.^143^ To address this, Qu *et al.*^144^ designed a conductive hydrogel based on GO for treating infected diabetic wounds. The incorporation of free charges and GO into the hydrogel network enhances its electrical conductivity to levels similar to that of human skin. This system shows excellent potential for use in bioelectronic signal transduction and wound healing (Fig. 8A). The bioelectrical stimulation directly affects the behaviour of macrophages, prompting a phenotypic shift from pro-inflammatory M1 to pro-regenerative M2 polarisation (Fig. 8B). This electrically driven immune modulation reduces TNF-α and IL-6 expression while increasing IL-10 secretion. In turn, this establishes a regenerative microenvironment that supports angiogenesis and tissue repair (Fig. 8C). These findings show that the injected conductive hydrogel effectively regulates macrophage polarisation, modulates the local immune microenvironment, supports angiogenesis, and significantly accelerates diabetic wound healing.

**Fig. 9:**
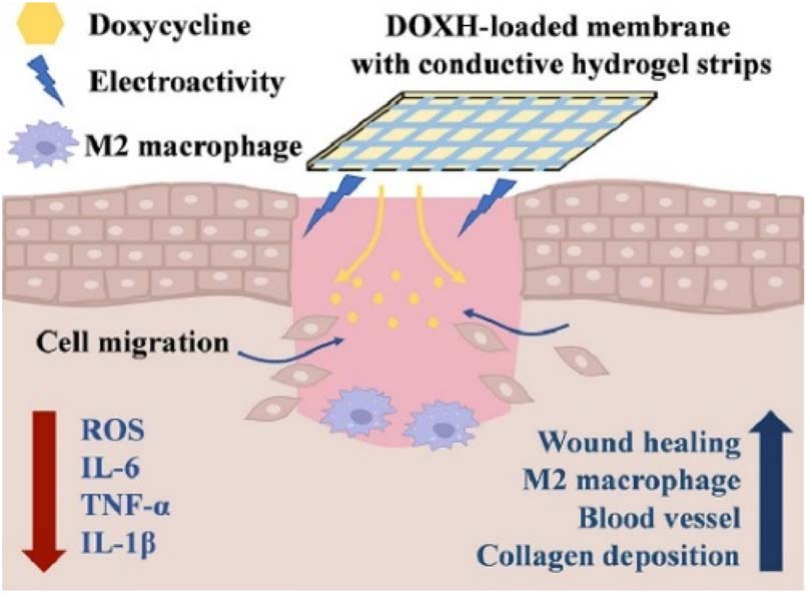
Treatment of diabetic wound with a composite dressing composed of conductive hydrogel strips and DOXH-loaded PFKU fibrous membrane accelerates wound healing by upregulating collagen deposition and neo-vascularisation. This figure was reproduced from ref. 54 with permission from Elsevier, copyright 2022.

Conductive hydrogel dressings can act as drug delivery platforms for sustained-release therapeutics in diabetic wounds. Cao *et al.*^54^ developed a dual-layer, multifunctional wound dressing. It consists of 3D-printed conductive hydrogel (GelMA-Bio-IL) strips and reactive polyurethane (PFKU) membranes loaded with doxorubicin hydrochloride (DOXH). This design enabled sustained local drug release while also scavenging ROS and using the conductive microenvironment to promote macrophage polarisation toward the M2 phenotype. Through these synergistic effects, the dressing effectively reduces chronic inflammation and creates a regenerative wound environment. This ultimately enhances collagen deposition and angiogenesis (Fig. 9).

Diabetic ulcers (DUs) are among the most severe and intractable complications of diabetes. Due to impaired nociception from diabetic neuropathy, minor injuries (*e.g.*, cuts, blisters, or burns) may develop into chronic DUs without being noticed. This increases the risk of amputation or life-threatening sepsis.

For antibiotic-loaded hydrogels, the burst release of antibiotics requires frequent dressing changes and raises the risk of antimicrobial resistance. To solve this issue, antimicrobial terbium ions (Tb^3+^) were incorporated into the hydrogel network through coordination bonding, enabling sustained release of the ions. When used with electrical stimulation in rat models of diabetic ulcers, the AZP-Tb hydrogel significantly accelerated wound healing by promoting both inflammatory and proliferative phases, which in turn sped up wound closure.^145^ Beyond inorganic ions, conductive biomaterials that include insulin, fibroblasts, or MSCs have also shown great potential for promoting tissue regeneration and functional recovery in diabetic wounds.^146^

### Joint wounds

Hydrogel wound dressings have gained increasing attention due to their ability to promote angiogenesis, thereby accelerating wound healing. Compared to wounds in static regions, those near joints face more complex biomechanical challenges.^147^ Because of frequent flexion and extension movements, conventional dressings often fail to maintain long-term and stable adhesion in joint areas, leading to displacement, wrinkling, or even secondary trauma. Additionally, the skin in these areas is exposed to high tensile stress and shear forces. This continuous mechanical stress slows wound healing and increases the risk of infection. Conductive hydrogels have emerged as promising options for joint wound repair given their excellent conformability, stretchability, NIR photothermal responsiveness, antimicrobial activity, biomechanical compatibility, and real-time sensing capabilities.

**Fig. 10:**
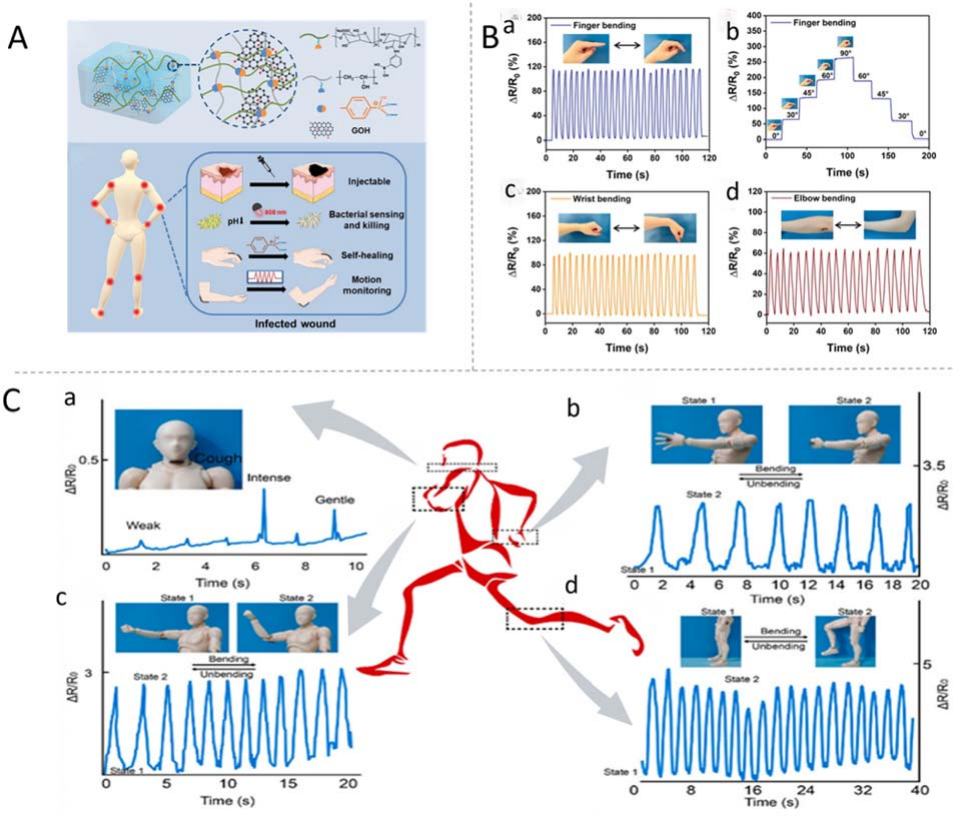
(A) Schematic of the crosslinked network, application and multi-functions of the Alg-PBA/PVA/GOH hydrogels. (B) Demonstration of the Alg-PBA/PVA/GOH hydrogel as a wearable sensor with strain-responsive conductivity. Relative resistance changes when monitoring bending of the (a) human finger; (b) index finger at different bending angles between 0° and 90°; (c) human wrist; and (d) human elbow. These figures were reproduced from ref. 148 with permission from Wiley, copyright 2024. (C) Movement sensing by attaching a strip of HSa (0.5 cm × 1 cm): (a) cough, (b) finger, (c) elbow, and (d) knee movement. The insets show photographs of the sensors. This figure was reproduced from ref. 149 with permission from Elsevier, copyright 2022.

Traditional hydrogel dressings are susceptible to disintegration and bacterial infection when used to manage joint wounds. To solve these issues, Zhou *et al.*^147^ developed a multifunctional hydrogel (Alg-PBA/PVA/GOH hydrogel) using polysaccharide biopolymers, PVA, and hydroxylated graphene. The structure of the hydrogel is stabilised by dynamic borate ester bonding and supramolecular interactions and offers rapid self-healing, injectability, conductivity, and motion monitoring capabilities (Fig. 10A). For use as a joint-wound dressing, the Alg-PBA/PVA/GOH hydrogel was integrated into a flexible strain sensor and applied to the index finger, wrist, and elbow joints (Fig. 10B). Its conductive network enables a sensitive response to joint bending. The sensor's electrical signal output increases with the bending angle, which allows real-time detection of the amplitude of joint movement. This function directly addresses the risk of wound re-tearing in dynamic joints by warning against excessive movement. Additionally, the hydrogel has strong antibacterial properties, achieved through regulating electrical signals and photothermal therapy. In vivo studies confirm that this conductive hydrogel effectively promotes joint-wound healing by tackling three key challenges related to joint wounds: disintegration of the dressing, infection, and movement-induced re-tearing. Furthermore, these findings highlight the hydrogel's significant potential as a multifunctional bioelectronic dressing, which can integrate detection, treatment, and management of infected joint wounds.

To address the unique challenges of joint wounds, which include dressing displacement caused by frequent movement, limited adaptability to irregular wound shapes, and the absence of real-time monitoring of the healing process. Chen *et al.*^39^ developed a novel polymeric conductive hydrogel (HSa). This hydrogel is formed by mixing a chitosan quaternary ammonium salt (HACC) and sodium alginate (SA). The key advantage of the HSa hydrogel is its conductivity, which comes from the free chloride ions (Cl^−^) in HACC. This conductivity directly supports the effective management of joint wounds. First, the resistance of the hydrogel shows a positive linear relationship with the deformation area, a property that enables reliable monitoring of joint movement and subtle muscular contractions. When applied to joints, it can detect resistance changes caused by different movements (*e.g.*, bending and stretching) and even coughs of varying intensity. As the amplitude of the movement increases, the resistance variations rise proportionally (Fig. 10C). This real-time monitoring function helps prevent excessive joint activity, which could otherwise lead to wound re-tearing. In turn, this addresses the issue of dynamic damage in joint wound care. Additionally, the hydrogel has excellent haemostatic properties, hemocompatibility, and cytocompatibility. Furthermore, its conductivity facilitates collagen deposition and vascularisation during wound repair, promoting effective healing.

## Challenges and future perspectives

Wound healing is a complex biological process. As biomedical science and materials science continue to develop and integrate, research and development related to new, efficient, intelligent, and microenvironment-adaptable hydrogels has created new avenues and opportunities for wound repair. Conductive hydrogels, which have electrical conductivity similar to that of human skin, can be used to support cellular activity. By regulating cellular signalling pathways, conductive hydrogels promote cell proliferation and migration, making them highly promising for use in wound dressings. However, the application of conductive hydrogels in wound dressings remains in its early stages, and their clinical implementation faces many challenges.

A balance must be struck between the biodegradability and conductivity of conductive hydrogels. These hydrogels typically consist of a hydrophilic polymer network combined with conductive components. However, most conventional systems prioritise conductivity over biodegradability, relying on non-degradable synthetic polymers (*e.g.*, PEDOT:PSS) or inorganic fillers. Converting these components into conductive oligomers (*e.g.*, short-chain aniline/pyrrole derivatives) by introducing cleavable bonds can improve the biodegradability, but this inherently compromises the conductivity.

Additionally, the conductivity of conductive hydrogels is affected by various factors, including pH, dopants, and complex wound environments. The long-term stability of their conductivity in wound applications has not yet been thoroughly studied.

The conductivity of conductive hydrogels changes significantly with pH variations, driven by protonation or deprotonation of their functional groups. Under acidic conditions, hydrogels containing basic groups (*e.g.*, –NH_2_) undergo protonation, enhancing their ionic conductivity. Conversely, under alkaline conditions, acidic groups (*e.g.*, –COOH) are ionised, altering the charge density and swelling behaviour.^172,173^ For instance, conductive hydrogels with PANI groups lose conductivity at pH > 4 due to deprotonation.^160^ In wound environments, the pH shift from acidic (early inflammatory phase) to neutral or alkaline (proliferative phase) directly affects the performance of conductive hydrogels.^174^

While doping agents improve the conductivity, they present stability challenges. Ion dopants migrate under electric fields, leading to reduced conductivity. Conductive polymers (*e.g.*, sulfated polypyrrole such as *p*-toluenesulfonic acid) enhance the stability but tend to aggregate in biological fluids.^175^ Carbon nanomaterials (*e.g.*, graphene, carbon nanotubes) provide conductive pathways but are prone to hydrolysis in wound exudate, resulting in decreased performance.^176^ During prolonged hydration, dopant leaching is a key factor impacting the stability. In simulated wound fluid environments, this leaching causes a drop in the conductivity of the hydrogel.^177^

The complex environment of wound surfaces also impacts the stability and conductivity of conductive hydrogels. Enzymatic degradation is a key factor in this context. Proteases in wound exudate can hydrolyse the protein matrix within hydrogels (*e.g.*, collagen, gelatin), disrupting the three-dimensional network and lowering the electrical conductivity of the hydrogels.^178^ Additionally, oxidative stress is particularly significant in chronic wounds like DUs. ROS in the wound oxidises conductive polymers in the hydrogel (*e.g.*, PEDOT), reducing its conductivity. Microbial activity, especially the formation of bacterial biofilms, also greatly affects the stability of hydrogels.^31^ Biofilms can alter the local pH of the wound environment and speed up degradation of the hydrogel matrix by secreting hydrolases, leading to more rapid conductivity loss.^153^ However, most current research focuses primarily on using hydrogels in the early stages of wound healing, with little attention paid to their performance in long-term healing. The lack of long-term *in vivo* conductivity data limits a full understanding of how the stability and conductivity of hydrogels change during extended wound healing.^179^

For the successful clinical translation of conductive hydrogel dressings, two key factors must be prioritised: long-term biocompatibility and regulatory approval. While conductive hydrogels show potential in short-term wound-healing studies, their long-term biological safety, performance characteristics, and interactions with surrounding tissues remain understudied. Furthermore, regulatory authorities require rigorous oversight of biocompatibility, toxicity testing, and long-term safety evaluations before approving hydrogels for clinical use. Thus, ensuring the long-term biocompatibility of conductive hydrogels and navigating the regulatory process are critical steps in promoting their widespread clinical application.

Currently, the lack of standardised evaluation metrics across different studies makes it difficult to draw meaningful comparisons and limits the reproducibility of research findings in the conductive-hydrogel field. Developing universal, standardised evaluation metrics is, therefore, essential. Such metrics would establish a reliable framework for comparing results, ensuring experimental consistency, and improving reproducibility.

The future development of conductive hydrogels will focus on integrating real-time monitoring of the wound environment (*e.g.*, pH levels, infection indicators, temperature, humidity, and bacterial load) with responsive control of electrical stimulation and drug release based on sensor data. This integration is expected to combine electrical stimulation with pharmacological therapy, photothermal treatment, and other synergistic strategies. The goal is to create safe, cost-effective, biodegradable, and low-toxicity conductive hydrogels that better meet clinical requirements for wound care.

Furthermore, using conductive materials in 3D printing could enable the fabrication of custom dressings tailored to individual wound shapes and complex topographies. This would reduce the risk of infection and support precision wound care. These advances hold promise for developing safe, cost-effective, biodegradable, and clinically relevant hydrogel-based therapies for personalised wound management.

## Conclusion

Compared to previous reviews, this work provides a focused evaluation of the critical applications of conductive hydrogels in wound dressings, specifically exploring their potential across various wound types. The review provides a comprehensive analysis of the electroactive mechanisms that drive wound healing, including how conductive hydrogels regulate cellular behaviours through electrical stimulation. Examples of such regulation include promoting fibroblast proliferation and migration, supporting vascular endothelial cell tubulogenesis, and modulating immune cell functions. The review also discusses how these hydrogels influence the expression of key growth factors and enhance local microcirculation, ultimately accelerating wound healing.

Furthermore, the review examines the unique design principles, functional performance requirements, and multifaceted mechanisms-of-action that make conductive hydrogels “smart” wound dressings in the dynamic and complex healing microenvironment. It also summarises the notable efficacy of these materials in preclinical animal models. Additionally, the review systematically outlines the key challenges that impede their clinical translation, providing a valuable reference for the future design and development of intelligent hydrogel-based wound dressings.

## Author contributions

Yun Lv: conceptualization, data curation, methodology, writing – original draft. Yuting Li: methodology, formal analysis. Yueshuai Pan: supervision, validation. Qianqian Li: data curation. Changfang Shi: visualization. Ruting Gu: supervision, writing – review & editing. Lili Wei: project administration, writing – review & editing.

## Conflicts of interest

There are no conflicts to declare.

## Data Availability

No primary research results, software or code are included, and no new data were generated or analysed as part of this review.
